# Against the Odds: A Novel Technique to Perform Cholangiography from a Percutaneous Approach through the Cystic Duct

**DOI:** 10.7759/cureus.3577

**Published:** 2018-11-12

**Authors:** Rebekah M Padilla, Paul C Hulsberg, Erik Soule, Taylor S Harmon, Erik Eadie, Preston Hood, Michael Shabandi, Jerry Matteo

**Affiliations:** 1 Interventional Radiology, Georgia Campus - Philadelphia College of Osteopathic Medicine, Suwanee, USA; 2 Interventional Radiology, University of Florida College of Medicine, Jacksonville, USA; 3 Interventional Radiology, The University of Texas Medical Branch, Galveston, USA

**Keywords:** acute cholecystitis, choledocholithiasis, cholelithiasis, steerable microcatheter, interventional radiology, biliary obstruction, percutaneous cholecystostomy, pancreatitis, wireless interventional procedure, percutaneous transhepatic cholangiography

## Abstract

Percutaneous cholangiography is typically performed via a transhepatic approach and is reserved for patients with contraindications to traditional cholangiogram imaging modalities. For those with suspected cholelithiasis or choledocholithiasis who cannot undergo magnetic resonance imaging for diagnosis, percutaneous cholecystostomy with cholangiogram is a viable option. Endoscopic retrograde cholangiopancreatography may also be precluded due to anatomic or obstructive limitations, in which case, percutaneous transhepatic cholangiography (PTC) may be indicated for diagnosis. PTC may be difficult in a patient with minimal biliary tree dilatation, or tortuous cystic duct anatomy. In cases such as these, a steerable microcatheter (SMC) may be utilized to enable or expedite PTC. The technique to traverse and catheterize the cystic duct and opacify the gallbladder, bile ducts, and duodenum utilizing an SMC is described. This report outlines a non-vascular application of the SMC resulting in a successful cholangiogram, with reduced operative time and thus reduced radiation exposure to the patient.

## Introduction

Percutaneous cholecystostomy with cholangiogram has been utilized and described since 1979 as a safe and effective diagnostic and treatment modality for patients who are poor surgical candidates [1]. Since then, percutaneous cholecystostomy has been used to relieve various biliary diseases causing blockage or obstruction in the biliary tree in which endoscopic retrograde cholangiogram (ERCP) has failed or surgery is contraindicated. Using percutaneous means to perform cholangiograms is typically completed via percutaneous transhepatic approach into the hepatic bile ducts. Once access is gained, contrast is injected, and fluoroscopy allows imaging of the biliary system. This can be challenging in a nondilated biliary system and non-percutaneous modalities are often attempted prior to a percutaneous transhepatic cholangiogram (PTC). Other special indications for percutaneous cholecystostomy include obstruction of the distal common bile duct [2]. The most common indications for percutaneous cholecystostomy are calculous and acalculous cholecystitis [3]. Additionally, percutaneous cholecystostomy has been shown to be an effective treatment for acute cholecystitis with severe comorbidities, allowing interval laparoscopic cholecystectomy with high success rates and lower morbidity [3,4]. Used in treatment of cholangitis and cholecystitis, the Tokyo guidelines recommend percutaneous cholecystostomy in moderate and severe forms cholecystitis [5,6].

Another modality to evaluate biliary obstruction in oral cholecystogram involves iodinated contrast media being administered orally followed by a meal. Radiographs are taken after contrast is excreted in the bile and choleliths are represented by filling defects. Oral cholecystogram is limited in sensitivity and specificity, and while useful to evaluate in an out-patient setting, is a lengthy procedure to wait on in an acute setting and may not be practical in a patient with acute obstruction. Cholescintigraphy is another useful tool, but is relatively contraindicated with known history of cholelithiasis, due to possible induction of biliary colic. Further, patients with contraindications to magnetic resonance cholangiopancreatography (MRCP), such as a pacemaker, or to ERCP, such as a Roux-en-Y gastric bypass, may be left with PTC as the only option.

Cholangiogram using percutaneous cholecystostomy may be facilitated with the increased maneuverability to aid in cannulation of tortuous cystic bile ducts. A steerable microcatheter (SMC) can be a viable option to traverse difficult anatomy or other obstructive diseases, as described for percutaneous nephrostomy, previously [7]. Moreover, using an SMC without using a guidewire can significantly shorten procedure time as well as reduce radiation time [8]. The case presented describes a technique utilizing an SMC to catheterize the cystic duct and perform diagnostic cholangiography of the common bile duct.

## Technical report

**Figure 1:**
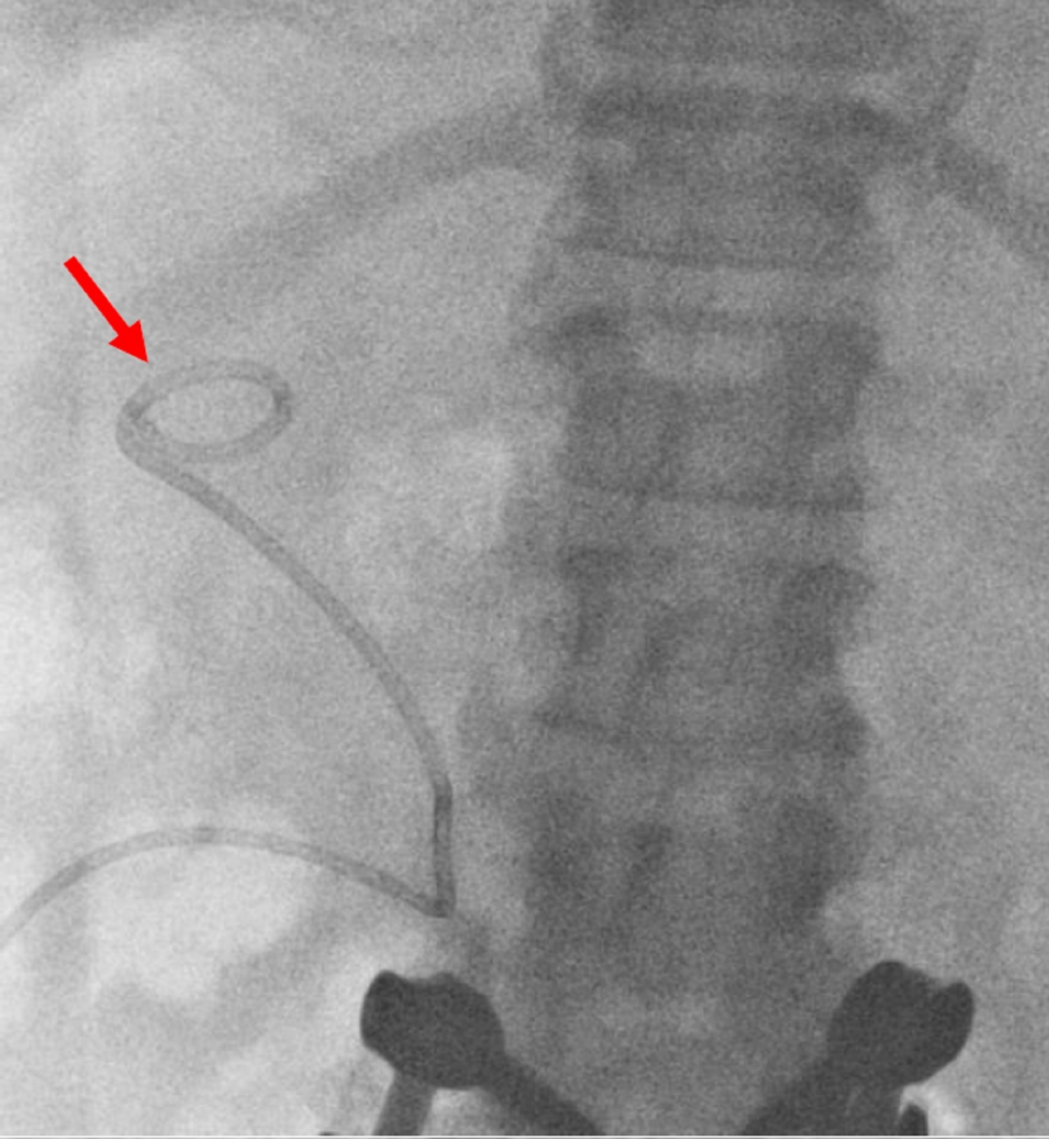
A cholecystostomy tube is seen in the gallbladder fossa (red arrow).

A 65-year-old African-American female presented to the emergency department with pancreatitis, elevated bilirubin, and history of calculous cholecystitis. Six weeks prior to current admission, a cholecystostomy was performed with planned laparoscopic cholecystectomy. The patient had contraindications to MRCP testing due to a pacemaker as well as contraindication to ERCP due to history of Roux-en-Y gastric bypass. Due to her history of cholelithiasis with pancreatitis and a rising bilirubin, there was clinical suspicion of choledocholithiasis, prompting need for cholangiography. Thus, diagnostic cholangiography was indicated and planned to be performed, with entry point utilizing the current cholecystostomy. Cholecystogram had been performed by introducing a bolus of contrast into the existing cholecystostomy access one week prior to admission and demonstrated the cystic duct to be obstructed. To avoid putting the patient through a lengthy and challenging PTC, an SMC was used to catheterize the cystic duct. Scout images demonstrated the drainage catheter in the gallbladder fossa (Figure 1).

**Figure 2:**
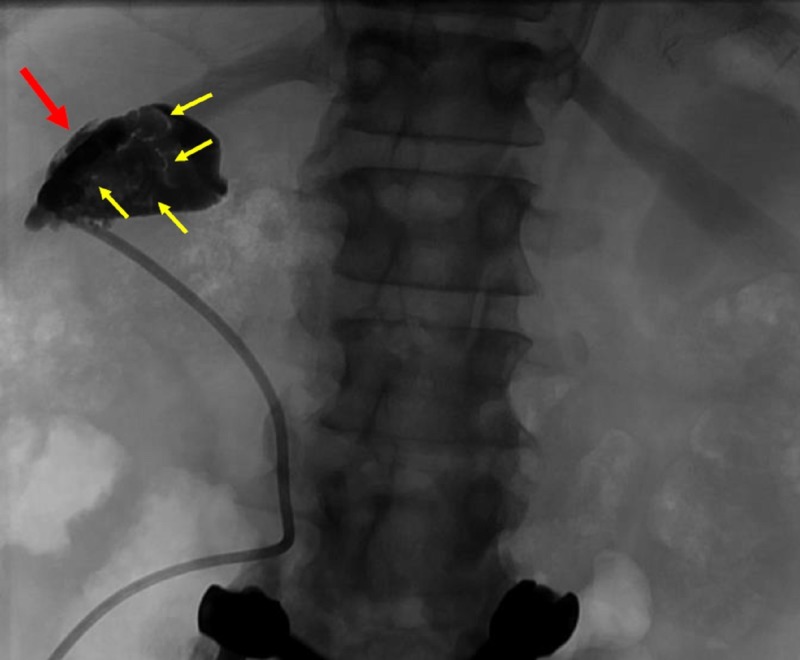
Contrast was injected into the gallbladder using a cholecystostomy tube in place (red arrow). Filling defects (yellow arrows) representing choleliths are visualized. No contrast reached the cystic duct.

Contrast was instilled through the patient’s current cholecystostomy tube. This demonstrated multiple filling defects representing choleliths without filling of the cystic duct (Figure 2).

**Figure 3:**
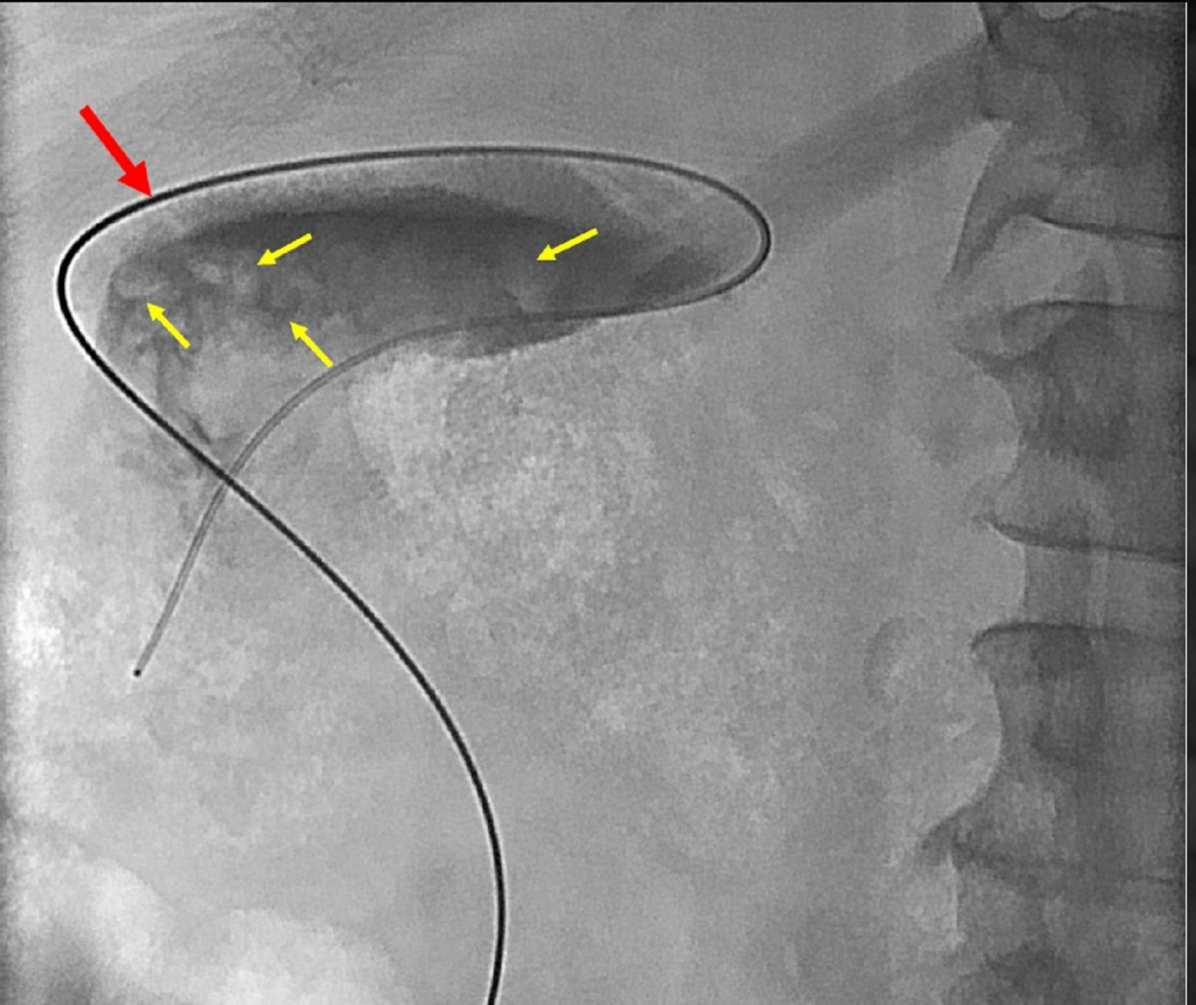
A Kumpe catheter was placed over a guidewire (red arrow) and exchanged for the cholecystostomy tube. Contrast was injected. Filling defects represent choleliths (yellow arrows). The cystic duct did not opacify.

A guidewire was advanced through the tube and coiled within the gallbladder. A five French Kumpe catheter was then advanced over the wire and used to catheterize the orifice of the cystic duct. However, contrast injection showed no opacification of the remainder of the cystic duct (Figure 3). While maintaining this guidewire in place, the previously placed tube was removed.

**Figure 4:**
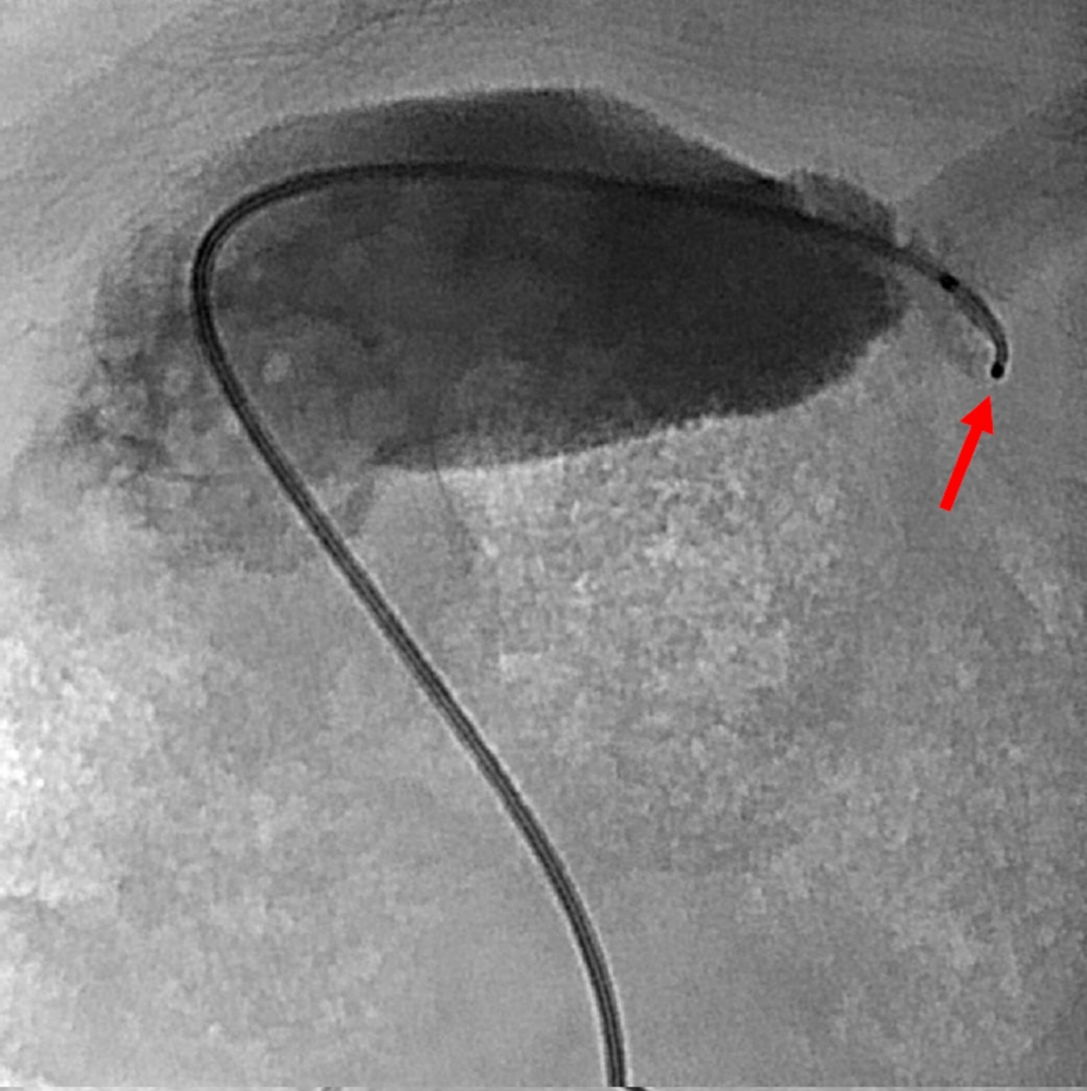
The steerable microcatheter tip depicted by the radiopaque markers (red arrow) was advanced to the cystic duct.

A SwiftNINJA SMC (Merit Medical, South Jordan, UT) was advanced through the five French catheter, and steering the flexible tip, the proximal cystic duct was quickly accessed and catheterized (Figure 4).

**Figure 5:**
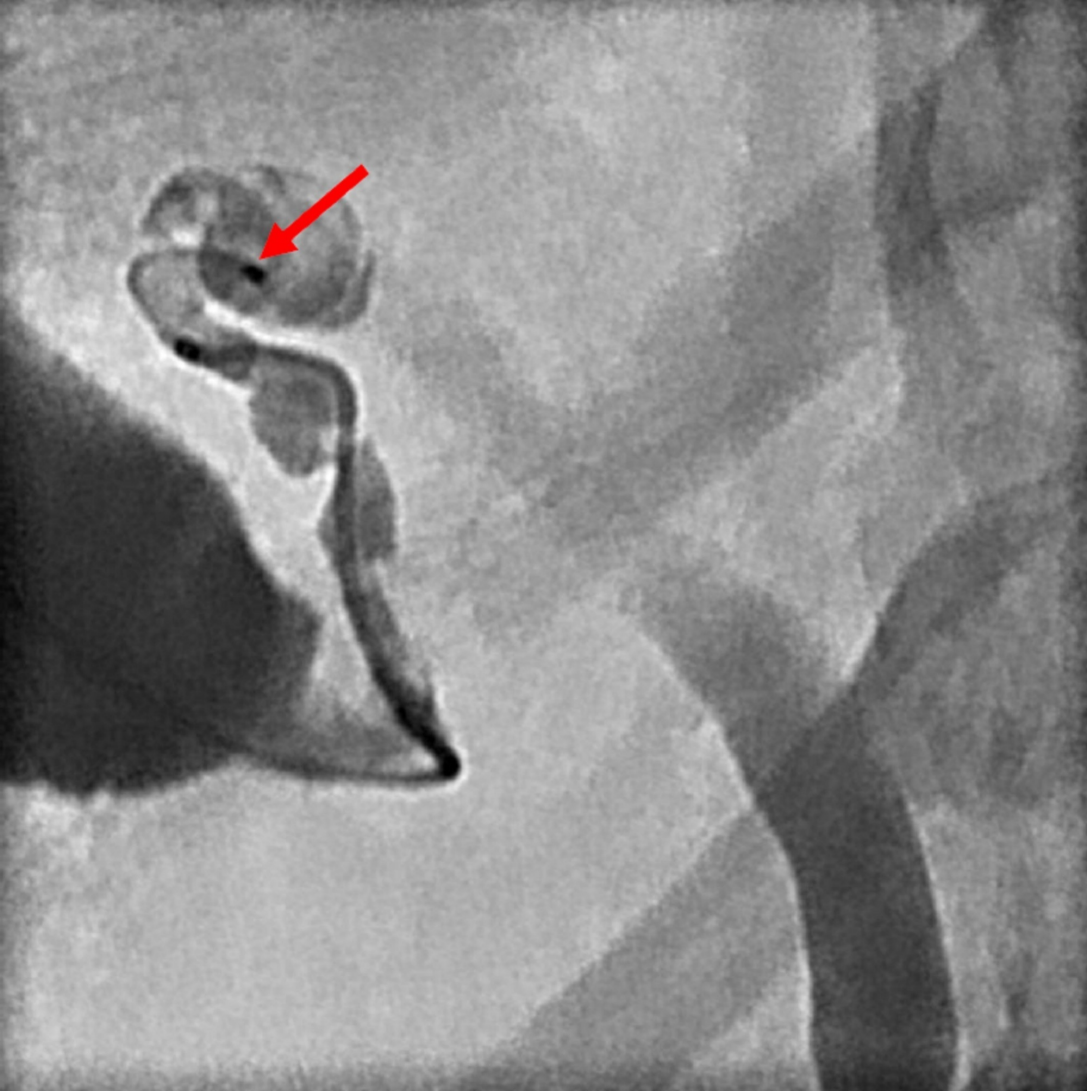
The microcatheter was then steered into a more distal position within the cystic duct (red arrow) and contrast was injected. Contrast opacified a tortuous cystic duct and progressed to fill the common bile duct.

Thereafter, the SMC was advanced midway through the cystic duct, by using the dial to adjust the angle of the tip and follow the anatomy seen on fluoroscopy. The cystic duct was opacified using intermittent contrast injection. Additional oblique views demonstrated the orifice of the cystic duct and common bile duct to be opacified (Figure 5).

**Figure 6:**
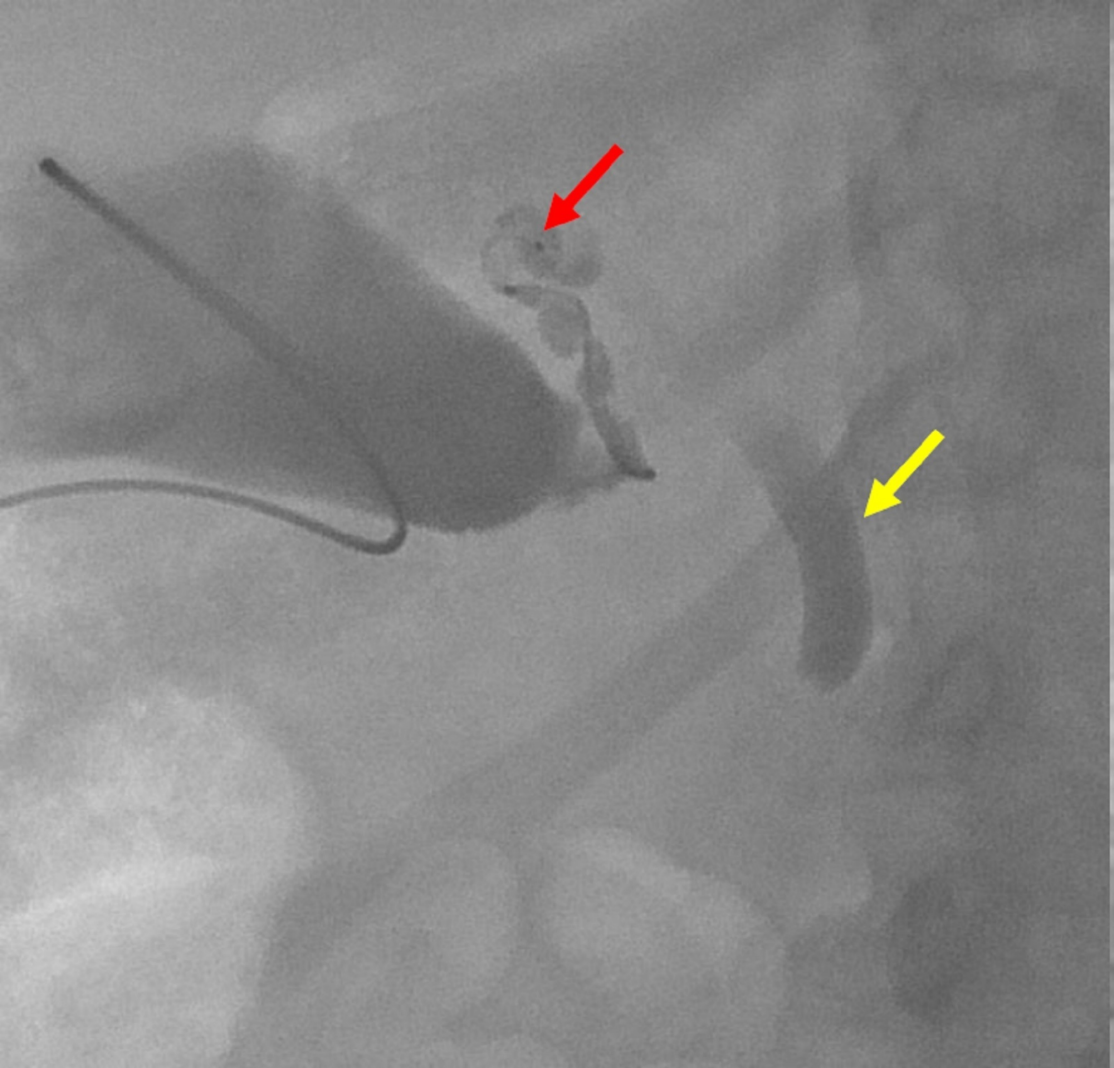
Contrast continued to be injected from the steerable microcatheter (red arrow) until the common bile duct was filled with contrast (yellow arrow).

No filling defects are seen within the cystic duct or common bile duct. Tapering of the distal common bile duct just before the sphincter of Oddi is seen in Figure 6, likely representing external compression, without evidence of choledocholithiasis.

**Figure 7:**
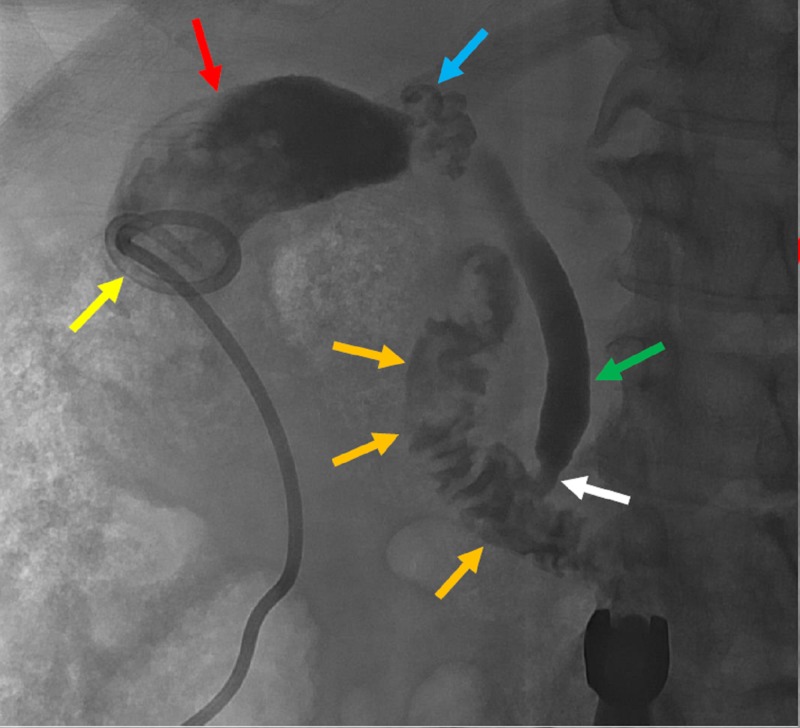
The microcatheter was exchanged for a pigtail catheter (yellow arrow) into the contrast filled gallbladder (red arrow). A successful cholangiogram demonstrated a tortuous cystic duct (blue arrow) and a patent common bile duct (green arrow) with tapering (white arrow), but without obstruction. Contrast was seen extending into the duodenum (orange arrows).

The SMC was removed, and a wire was advanced through the five French catheter and coiled within the gallbladder. A new drainage catheter was advanced over the wire and the distal tip was formed within the gallbladder lumen. Contrast material instilled through this new tube and imaging obtained showed appropriate position of the drainage catheter. Final imaging showed contrast filling the cystic duct, common bile duct, and parts of the duodenum (Figure 7).

The drainage catheter was secured in place with a needle-less fixation device. The patient tolerated the procedure well without immediate or short-term complications.

## Discussion

Laparoscopic cholecystectomy is the standard of care for cholecystitis. Percutaneous cholecystectomy has become a valuable bridge to surgery to reduce inflammation while decompressing the gallbladder, reducing adhesions, and showing a decreased conversion to open surgery [7, 8]. Interventional radiology plays an important role to improve the process in terms of technique and patient safety. PTC is a viable option to image the biliary system for obstructing stones when contraindications to both MRCP and ERCP are noted.

When a cholecystectomy tube is in place and further investigation of the remainder of the biliary system is desired, a cholangiogram via the existing catheter is preferred. However, if cholelithiasis or tortuous cystic duct anatomy precludes contrast opacification of the biliary ductal system, an SMC may provide a practical method of performing cholangiography which may not be otherwise technically possible. The reported technique presented shows success notwithstanding a gallbladder packed with gallstones, making passage difficult using an ordinary catheter. However, these obstructions were easily bypassed with a non-vascular application of an SMC.

Utilization of an SMC without a guidewire access is feasible and safe [9,10]. Additionally, the extra-vascular use of an SMC to navigate difficult obstructions shows promise for patients with obstructive renal calculi, or in this case, cholelithiasis [9].

Percutaneous transcholecystic access to the common bile duct has been previously described, however, navigating of a tortuous cystic duct may be impossible using a guidewire [11]. Utilizing the SMC to perform cystic duct cannalation is a novel strategy to approach patients with biliary obstruction. When the bile duct is not dilated, a lengthy and tedious hepatic duct catheterization can be avoided. By limiting exchanges, this approach decreases the risks for bile. Limiting catheterization attempts reduce risk of infection and bleeding. Using the cholecystostomy access a patient already has, multiple needle punctures through the liver and a potentially painful PTC can be avoided. Although the cystic duct orifice may be obstructed in patients with cholelithiasis, the SMC is agile and small enough to traverse the rocky path. This procedure is faster, resulting in less radiation exposure, and less patient discomfort. Using an SMC is a solution to many technical hurdles seen in patients with contraindications to MRCP and ERCP with need for evaluation of the common bile duct and cystic duct.

## Conclusions

As technology advances, naturally, so does medical equipment and techniques. The SMC may be a viable option for percutaneous cholecystostomy and cholangiogram not technically possible due to anatomy, obstruction, or medical contraindications. Excellent technical outcome, imaging, shorter procedure time were observed using this technique, resulting in lower radiation exposure to patients and staff. Using the SMC in biliary applications may decrease procedure time and increase patient safety and satisfaction.
